# State-Specific Prevalence of Tobacco Product Use Among Adults — United States, 2014–2015

**DOI:** 10.15585/mmwr.mm6703a3

**Published:** 2018-01-26

**Authors:** Satomi Odani, Brian S. Armour, Corinne M. Graffunder, Gordon Willis, Anne M. Hartman, Israel T. Agaku

**Affiliations:** ^1^Office on Smoking and Health, National Center for Chronic Disease Prevention and Health Promotion, CDC; ^2^Oak Ridge Institute for Science and Education (ORISE) Fellow; ^3^Behavioral Research Program, Division of Cancer Control and Population Sciences, National Cancer Institute, Rockville, Maryland.

Despite recent declines in cigarette smoking prevalence, the tobacco product landscape has shifted to include emerging tobacco products* (*1*,*2*). Previous research has documented adult use of smokeless tobacco and cigarettes by state (*3*); however, state-specific data on other tobacco products are limited. To assess tobacco product use in the 50 U.S. states and the District of Columbia (DC), CDC and the National Cancer Institute analyzed self-reported use of six tobacco product types: cigarettes, cigars, regular pipes, water pipes, electronic cigarettes (e-cigarettes), and smokeless tobacco products among adults aged ≥18 years using data from the 2014–2015 Tobacco Use Supplement to the Current Population Survey (TUS-CPS). Prevalence of ever-use of any tobacco product ranged from 27.0% (Utah) to 55.4% (Wyoming). Current (every day or some days) use of any tobacco product ranged from 10.2% (California) to 27.7% (Wyoming). Cigarettes were the most common currently used tobacco product in all states and DC. Among current cigarette smokers, the proportion who currently used one or more other tobacco products ranged from 11.5% (Delaware) to 32.3% (Oregon). Differences in tobacco product use across states underscore the importance of implementing proven population-level strategies to reduce tobacco use and expanding these strategies to cover all forms of tobacco marketed in the United States. Such strategies could include comprehensive smoke-free policies, tobacco product price increases, anti-tobacco mass media campaigns, and barrier-free access to clinical smoking cessation resources (*1*,*4*). 

The 2014–15 TUS-CPS was a household-based survey of adults aged ≥18 years in the 50 U.S. states and DC (*5*). A total of 163,920 respondents participated (response rate = 54.2%).^†^ Six tobacco product types were assessed: cigarettes, cigars (including regular cigars, cigarillos, or little filtered cigars), regular pipes, water pipes, e-cigarettes, and smokeless tobacco products (including moist snuff, dip, spit, chew tobacco, snus, or dissolvable tobacco).

For all tobacco product types except cigarettes,^§^ ever-users were defined as persons who had used the respective products one or more times during their lifetime; current users were persons who reported ever-use and who used the respective products “every day” or “some days” at the time of survey. Ever cigarette smokers were defined as persons who had smoked 100 or more cigarettes during their lifetime; current cigarette smokers were persons who reported ever cigarette smoking and smoked “every day” or “some days” at the time of survey. Any tobacco product use was defined as use of any of the six assessed tobacco products,^¶^ and any combustible tobacco product use was defined as any use of cigarettes, cigars, regular pipes, or water pipes.** Data were weighted to yield state-representative estimates. Prevalence estimates with relative standard errors ≥30% were suppressed.

Prevalence of ever-use ranged from 27.0% (Utah) to 55.4% (Wyoming) for any tobacco product, from 25.8% (Utah) to 53.2% (Maine) for any combustible tobacco product, from 22.0% (Utah) to 44.3% (Maine) for cigarettes, from 10.6% (Utah) to 26.3% (Oregon) for cigars, from 4.3% (Delaware) to 14.2% (Wyoming) for e-cigarettes, from 2.7% (New Jersey) to 20.5% (Wyoming) for smokeless tobacco, from 3.2% (New Jersey) to 12.0% (Oregon) for regular pipes, and from 1.5% (Arkansas) to 16.7% (DC) for water pipes (Table 1).

**TABLE 1:** Prevalence of ever-use of any tobacco product, combustible tobacco and six tobacco products types among U.S. adults aged ≥18 years,* by state and tobacco product type — Tobacco Use Supplement to the Current Population Survey, United States, 2014–2015

State	Any tobacco^†^	Combustible tobacco^§^	Cigarettes^¶^	Cigars**	Regular pipe**	Water pipe**	Electronic cigarette**	Smokeless tobacco**
	% (95% CI)	% (95% CI)	% (95% CI)	% (95% CI)	% (95% CI)	% (95% CI)	% (95% CI)	% (95% CI)
Alabama	41.8 (39.6–43.9)	39.3 (37.2–41.4)	34.3 (32.3–36.3)	15.3 (13.7–16.9)	5.6 (4.6–6.6)	2.0 (1.3–2.7)	10.8 (9.3–12.2)	9.8 (8.4–11.3)
Alaska	51.5 (49.0–54.0)	49.1 (46.6–51.6)	39.2 (36.8–41.6)	24.3 (22.2–26.4)	9.1 (7.7–10.5)	6.6 (5.3–7.8)	11.7 (10.0–13.4)	15.0 (13.3–16.8)
Arizona	39.5 (37.4–41.7)	38.5 (36.4–40.7)	30.2 (28.3–32.2)	17.9 (16.1–19.6)	6.7 (5.7–7.8)	6.1 (5.0–7.3)	9.5 (8.1–10.9)	5.8 (4.8–6.8)
Arkansas	47.3 (45.1–49.4)	44.4 (42.2–46.5)	41.3 (39.2–43.4)	14.8 (13.3–16.3)	5.4 (4.5–6.3)	1.5 (0.9–2.0)	9.3 (8.1–10.6)	12.0 (10.5–13.4)
California	33.3 (32.4–34.2)	32.6 (31.8–33.5)	23.9 (23.1–24.6)	15.2 (14.5–15.8)	4.5 (4.1–4.9)	6.3 (5.8–6.8)	6.1 (5.7–6.6)	4.2 (3.8–4.6)
Colorado	49.4 (47.2–51.6)	47.7 (45.5–49.9)	35.8 (33.7–37.9)	24.2 (22.3–26.1)	8.3 (7.1–9.5)	7.6 (6.4–8.9)	9.9 (8.5–11.3)	13.1 (11.6–14.6)
Connecticut	43.5 (41.1–45.8)	42.6 (40.3–45.0)	35.6 (33.4–37.8)	18.7 (16.9–20.5)	6.1 (5.1–7.2)	4.7 (3.7–5.8)	6.9 (5.6–8.2)	4.8 (3.8–5.9)
Delaware	37.2 (35.1–39.4)	36.5 (34.4–38.7)	31.8 (29.8–33.8)	11.8 (10.4–13.3)	4.3 (3.4–5.2)	2.5 (1.7–3.2)	4.3 (3.3–5.2)	3.3 (2.5–4.1)
District of Columbia	46.9 (45.0–48.9)	46.3 (44.4–48.2)	28.6 (27.0–30.3)	22.5 (20.9–24.1)	5.7 (4.8–6.5)	16.7 (15.3–18.2)	7.6 (6.6–8.6)	4.9 (4.1–5.7)
Florida	34.9 (33.7–36.1)	34.2 (33.0–35.5)	30.2 (29.0–31.3)	12.0 (11.2–12.9)	3.8 (3.3–4.3)	3.0 (2.5–3.5)	5.8 (5.2–6.5)	3.4 (3.0–3.9)
Georgia	36.9 (35.2–38.6)	35.3 (33.6–37.0)	29.5 (27.9–31.1)	14.9 (13.6–16.1)	4.8 (4.0–5.5)	3.5 (2.8–4.2)	7.8 (6.8–8.8)	7.6 (6.6–8.5)
Hawaii	33.3 (31.0–35.5)	31.5 (29.3–33.7)	26.8 (24.7–28.9)	13.7 (12.0–15.3)	4.7 (3.7–5.7)	3.5 (2.5–4.4)	9.1 (7.5–10.6)	5.2 (4.1–6.2)
Idaho	42.5 (40.3–44.6)	41.3 (39.2–43.4)	32.3 (30.3–34.3)	21.0 (19.2–22.8)	7.5 (6.3–8.6)	5.5 (4.4–6.6)	10.3 (8.9–11.6)	10.5 (9.2–11.8)
Illinois	42.0 (40.5–43.5)	41.1 (39.6–42.6)	32.6 (31.2–34.0)	17.9 (16.8–19.1)	5.3 (4.7–6.0)	4.8 (4.1–5.6)	8.3 (7.4–9.1)	6.4 (5.6–7.1)
Indiana	49.7 (47.7–51.8)	48.5 (46.5–50.6)	40.2 (38.2–42.2)	21.4 (19.7–23.0)	7.1 (6.1–8.1)	4.7 (3.7–5.7)	10.6 (9.3–12.0)	9.2 (8.0–10.4)
Iowa	51.8 (49.6–54.0)	49.2 (46.9–51.4)	39.0 (36.9–41.2)	24.9 (22.9–26.8)	8.9 (7.6–10.2)	4.7 (3.7–5.8)	10.5 (9.0–11.9)	13.7 (12.2–15.3)
Kansas	46.3 (44.1–48.4)	44.1 (42.0–46.2)	35.3 (33.3–37.4)	21.0 (19.2–22.7)	7.1 (6.0–8.1)	5.3 (4.2–6.3)	12.3 (10.8–13.8)	11.2 (9.8–12.6)
Kentucky	49.4 (47.2–51.5)	47.3 (45.2–49.4)	41.8 (39.7–43.9)	17.5 (15.8–19.1)	6.6 (5.6–7.7)	3.1 (2.2–4.0)	11.0 (9.6–12.4)	11.3 (9.9–12.7)
Louisiana	38.5 (36.6–40.5)	36.9 (34.9–38.8)	32.7 (30.8–34.5)	12.2 (10.9–13.6)	4.1 (3.3–4.9)	2.1 (1.5–2.7)	8.3 (7.2–9.5)	6.9 (5.9–8.0)
Maine	54.1 (51.8–56.4)	53.2 (51.0–55.5)	44.3 (42.0–46.5)	23.9 (22.0–25.9)	10.8 (9.5–12.2)	4.7 (3.6–5.8)	9.7 (8.3–11.2)	8.3 (7.0–9.7)
Maryland	38.7 (36.7–40.7)	38.3 (36.3–40.3)	28.6 (26.7–30.4)	17.1 (15.6–18.7)	6.0 (5.0–7.0)	5.6 (4.6–6.7)	7.1 (6.0–8.3)	3.7 (2.9–4.5)
Massachusetts	41.9 (40.0–43.9)	41.4 (39.4–43.4)	32.5 (30.7–34.3)	17.4 (15.8–18.9)	5.5 (4.6–6.4)	6.2 (5.1–7.3)	6.6 (5.5–7.7)	3.4 (2.6–4.1)
Michigan	48.3 (46.6–50.0)	47.4 (45.7–49.1)	38.3 (36.7–39.9)	20.7 (19.3–22.1)	7.8 (6.9–8.7)	6.2 (5.3–7.2)	10.8 (9.6–11.9)	8.0 (7.0–8.9)
Minnesota	50.0 (48.0–51.9)	48.3 (46.4–50.3)	37.2 (35.3–39.0)	22.6 (20.9–24.2)	8.3 (7.2–9.3)	5.2 (4.3–6.2)	9.9 (8.7–11.1)	11.4 (10.2–12.7)
Mississippi	39.9 (37.9–41.9)	37.1 (35.1–39.0)	32.9 (31.0–34.8)	12.7 (11.3–14.0)	3.9 (3.1–4.6)	1.9 (1.3–2.4)	8.1 (7.0–9.3)	10.1 (8.8–11.4)
Missouri	49.0 (46.9–51.1)	47.2 (45.1–49.3)	39.3 (37.3–41.3)	20.5 (18.8–22.2)	7.3 (6.3–8.4)	4.0 (3.1–5.0)	9.9 (8.6–11.2)	9.8 (8.5–11.1)
Montana	50.7 (48.6–52.8)	47.9 (45.8–50.0)	36.5 (34.5–38.5)	24.2 (22.3–26.0)	10.2 (8.9–11.5)	5.6 (4.4–6.7)	9.6 (8.2–10.9)	16.0 (14.4–17.6)
Nebraska	46.9 (44.7–49.2)	45.0 (42.7–47.2)	36.3 (34.2–38.5)	21.1 (19.3–23.0)	6.2 (5.1–7.3)	4.4 (3.3–5.4)	11.8 (10.3–13.4)	10.5 (9.1–12.0)
Nevada	38.0 (35.8–40.2)	37.3 (35.2–39.5)	30.8 (28.8–32.8)	13.6 (12.1–15.1)	4.4 (3.6–5.3)	6.9 (5.6–8.1)	9.1 (7.7–10.4)	4.6 (3.6–5.5)
New Hampshire	49.6 (47.5–51.6)	49.0 (47.0–51.1)	41.5 (39.5–43.5)	20.0 (18.4–21.7)	7.7 (6.7–8.8)	4.6 (3.6–5.6)	7.8 (6.6–8.9)	6.5 (5.4–7.6)
New Jersey	34.1 (32.3–35.9)	33.8 (32.0–35.6)	28.9 (27.2–30.6)	11.4 (10.2–12.6)	3.2 (2.5–3.8)	2.6 (1.9–3.2)	5.1 (4.2–6.0)	2.7 (2.1–3.3)
New Mexico	38.8 (36.6–41.0)	37.6 (35.4–39.7)	31.3 (29.2–33.3)	14.8 (13.2–16.4)	4.5 (3.7–5.4)	3.5 (2.6–4.3)	7.3 (6.2–8.5)	5.9 (4.9–7.0)
New York	38.2 (36.8–39.5)	37.6 (36.3–39.0)	31.0 (29.8–32.2)	14.3 (13.4–15.3)	4.4 (3.9–4.9)	4.7 (4.1–5.4)	7.3 (6.6–8.1)	3.9 (3.3–4.4)
North Carolina	43.9 (42.1–45.7)	41.5 (39.7–43.3)	34.5 (32.8–36.2)	16.3 (14.8–17.7)	6.1 (5.2–7.1)	4.4 (3.6–5.3)	8.9 (7.8–10.1)	8.2 (7.1–9.2)
North Dakota	51.3 (49.1–53.5)	48.2 (45.9–50.4)	39.1 (36.9–41.2)	23.3 (21.4–25.2)	8.1 (6.9–9.4)	5.4 (4.2–6.5)	9.7 (8.4–11.1)	17.5 (15.7–19.2)
Ohio	50.2 (48.6–51.8)	48.7 (47.1–50.2)	39.6 (38.1–41.1)	22.2 (20.9–23.5)	7.3 (6.6–8.1)	4.5 (3.7–5.3)	11.2 (10.1–12.2)	9.6 (8.6–10.6)
Oklahoma	46.1 (44.0–48.2)	44.1 (42.0–46.3)	38.6 (36.5–40.7)	17.3 (15.7–19.0)	5.6 (4.7–6.5)	2.7 (2.0–3.5)	12.0 (10.6–13.4)	12.0 (10.6–13.4)
Oregon	49.7 (47.6–51.9)	48.3 (46.2–50.5)	37.6 (35.6–39.7)	26.3 (24.3–28.2)	12.0 (10.5–13.4)	6.4 (5.3–7.6)	10.1 (8.7–11.5)	11.7 (10.3–13.2)
Pennsylvania	48.4 (46.8–50.0)	46.9 (45.3–48.5)	38.5 (37.0–39.9)	20.5 (19.3–21.8)	7.1 (6.4–7.9)	4.2 (3.5–4.9)	9.1 (8.2–10.1)	9.1 (8.2–10.0)
Rhode Island	41.5 (39.0–44.0)	41.1 (38.6–43.5)	34.1 (31.8–36.4)	16.0 (14.1–17.8)	5.8 (4.7–6.8)	5.4 (4.0–6.7)	6.2 (4.9–7.5)	3.6 (2.6–4.6)
South Carolina	41.7 (39.7–43.8)	40.4 (38.4–42.4)	34.8 (32.9–36.7)	16.2 (14.7–17.8)	6.5 (5.4–7.5)	3.2 (2.4–4.0)	8.4 (7.2–9.6)	7.0 (6.0–8.0)
South Dakota	53.0 (50.7–55.3)	50.0 (47.7–52.3)	41.5 (39.2–43.8)	23.4 (21.4–25.4)	7.3 (6.1–8.4)	5.7 (4.5–6.9)	11.3 (9.7–12.8)	15.1 (13.4–16.8)
Tennessee	45.0 (43.0–47.0)	43.0 (41.1–45.0)	36.5 (34.6–38.4)	17.6 (16.1–19.1)	7.0 (6.0–7.9)	3.3 (2.6–4.1)	10.7 (9.4–11.9)	10.0 (8.8–11.1)
Texas	37.5 (36.3–38.6)	35.6 (34.5–36.7)	28.2 (27.2–29.2)	14.9 (14.1–15.7)	5.0 (4.5–5.5)	4.7 (4.1–5.2)	8.3 (7.6–8.9)	7.2 (6.6–7.8)
Utah	27.0 (25.0–29.0)	25.8 (23.8–27.8)	22.0 (20.1–23.9)	10.6 (9.2–12.1)	4.3 (3.4–5.3)	5.0 (3.9–6.0)	8.8 (7.4–10.1)	5.6 (4.6–6.7)
Vermont	52.6 (50.6–54.7)	51.7 (49.7–53.8)	42.8 (40.7–44.8)	21.9 (20.2–23.7)	11.1 (9.7–12.4)	5.1 (4.0–6.1)	8.7 (7.4–10.0)	8.7 (7.4–9.9)
Virginia	43.9 (42.1–45.7)	42.8 (41.0–44.7)	32.7 (31.0–34.4)	18.7 (17.3–20.1)	6.5 (5.6–7.4)	6.9 (5.9–7.9)	8.9 (7.8–10.0)	7.9 (6.9–8.9)
Washington	47.7 (45.8–49.6)	46.3 (44.4–48.2)	35.4 (33.7–37.2)	24.7 (23.0–26.3)	9.3 (8.2–10.4)	6.7 (5.7–7.7)	10.8 (9.6–12.0)	10.5 (9.4–11.7)
West Virginia	50.2 (48.1–52.3)	46.5 (44.4–48.6)	40.4 (38.3–42.4)	17.9 (16.1–19.6)	7.2 (6.0–8.3)	2.5 (1.7–3.3)	10.3 (9.0–11.7)	14.5 (12.9–16.1)
Wisconsin	50.2 (48.2–52.2)	48.9 (46.9–51.0)	37.8 (35.9–39.7)	24.4 (22.6–26.1)	7.5 (6.4–8.6)	4.3 (3.4–5.3)	9.2 (8.0–10.4)	10.0 (8.8–11.3)
Wyoming	55.4 (53.2–57.6)	51.3 (49.2–53.5)	40.6 (38.5–42.8)	23.9 (22.0–25.8)	9.9 (8.5–11.3)	6.9 (5.5–8.3)	14.2 (12.6–15.8)	20.5 (18.7–22.4)

**Abbreviation**: CI = confidence interval.

* n = 163,920. Data were weighted to adjust for nonresponse and to yield representative estimates at the state level.

^†^ Persons who reported ever use of at least one of the six tobacco products assessed (cigarettes, cigars, regular pipe, water pipe, e-cigarette, and smokeless tobacco).

^§^ Persons who reported having used cigarettes, cigars, regular pipe, or water pipe at least once during their lifetime.

^¶^ Persons who reported having smoked ≥100 cigarettes during their lifetime.

** Persons who reported having used the respective product at least once during their lifetime. Cigars includes regular cigars, cigarillos, or little filtered cigars. Smokeless tobacco includes moist snuff, dip, spit, chew tobacco, snus, or dissolvable tobacco.

In all states, cigarettes were the most commonly ever-used tobacco products, followed by cigars. The third most commonly reported ever-used product was e-cigarettes in 32 states (range for those states = 5.1% in New Jersey to 11.8% in Nebraska); smokeless tobacco in 14 states (9.1% in Pennsylvania to 20.5% in Wyoming); regular pipes in Delaware (4.3%), Maine (10.8%), and Vermont (11.1%); and water pipes in California (6.3%) and DC (16.7%).

Prevalence of current use of any tobacco product ranged from 10.2% (California) to 27.7% (Wyoming) (Table 2). Among respondents who had ever used any tobacco product, the proportion who were current users of any tobacco product ranged from 30.7% (California) to 57.7% (Mississippi) (not presented in Tables). Current use of any combustible tobacco product ranged from 8.9% (Utah) to 23.1% (West Virginia). Among respondents who had ever used any combustible tobacco product, the proportion who were current combustible tobacco product users ranged from 28.6% (California) to 53.0% (Mississippi). Current cigarette smoking prevalence ranged from 8.0% (Utah) to 21.7% (West Virginia); among ever cigarette smokers, the proportion who were current cigarette smokers ranged from 33.9% (California) to 57.3% (Louisiana). Prevalence of current cigar use ranged from 1.0% (Utah) to 3.5% (Alaska); among respondents who had ever smoked cigars, the proportion who were current cigar smokers ranged from 8.1% (Vermont) to 20.0% (New Jersey). The prevalence of current e-cigarette use ranged from 1.3% (Delaware) to 4.4% (Wyoming); among e-cigarette ever-users, the proportion who were current e-cigarette users ranged from 16.6% (DC) to 40.0% (Rhode Island). The prevalence of current smokeless tobacco use ranged from 0.6% (New York) to 6.4% (Wyoming); among respondents who had ever used smokeless tobacco, the proportion who were current smokeless tobacco users ranged from 6.7% (Maine) to 36.1% (Mississippi). The prevalence of current water pipe smoking prevalence ranged from 0.4% (Florida) to 1.9% (DC); among respondents who had ever smoked water pipes, the proportion who were current water pipe smokers ranged from 0.0% (Arkansas) and Oklahoma to 21.2% (Rhode Island). Finally, the prevalence of current regular pipe smoking ranged from 0.2% (Florida), to 1.0% (Oregon); among those who had ever smoked a regular pipe, the proportion who were current regular pipe smokers ranged from 2.9% (Georgia) to 13.0% (Utah).

**TABLE 2:** Prevalence of current use of any tobacco product, combustible tobacco and six tobacco products types among adults aged ≥18 years,* by state and tobacco product type — Tobacco Use Supplement to the Current Population Survey, United States, 2014–2015

State	Any tobacco^†^	Combustible tobacco^§^	Cigarettes^¶^	Cigars**	Regular pipe**	Water pipe**	Electronic cigarette**	Smokeless tobacco**
	% (95% CI)	% (95% CI)	% (95% CI)	% (95% CI)	% (95% CI)	% (95% CI)	% (95% CI)	% (95% CI)
Alabama	23.1 (21.2–24.9)	19.7 (17.9–21.5)	18.2 (16.5–19.9)	2.9 (2.2–3.7)	–^††^	–^††^	3.8 (3.0–4.6)	3.3 (2.5–4.0)
Alaska	21.4 (19.4–23.5)	18.5 (16.5–20.4)	16.2 (14.3–18.0)	3.5 (2.5–4.5)	–^††^	–^††^	2.4 (1.7–3.2)	3.5 (2.5–4.4)
Arizona	14.4 (12.9–15.9)	13.0 (11.5–14.5)	11.9 (10.5–13.3)	1.5 (0.9–2.0)	–^††^	1.1 (0.5–1.6)	2.4 (1.7–3.1)	0.9 (0.5–1.4)
Arkansas	24.0 (22.2–25.8)	20.4 (18.7–22.1)	20.0 (18.3–21.7)	1.9 (1.3–2.5)	–^††^	–^††^	2.8 (2.0–3.5)	4.0 (3.1–4.8)
California	10.2 (9.6–10.8)	9.4 (8.8–9.9)	8.0 (7.5–8.5)	1.7 (1.4–1.9)	0.2 (0.1–0.3)	0.6 (0.5–0.8)	1.4 (1.1–1.6)	0.6 (0.4–0.8)
Colorado	16.9 (15.2–18.6)	14.9 (13.3–16.5)	13.1 (11.6–14.6)	2.1 (1.5–2.7)	–^††^	0.8 (0.3–1.3)	2.6 (1.8–3.3)	1.8 (1.2–2.3)
Connecticut	15.4 (13.7–17.1)	14.3 (12.7–16.0)	12.3 (10.8–13.9)	3.0 (2.1–3.8)	–^††^	–^††^	2.3 (1.6–3.0)	–^††^
Delaware	15.2 (13.6–16.8)	14.3 (12.8–15.9)	13.3 (11.8–14.8)	1.9 (1.2–2.5)	–^††^	–^††^	1.3 (0.8–1.8)	–^††^
District of Columbia	15.8 (14.4–17.3)	15.5 (14.1–16.9)	12.2 (10.9–13.4)	3.0 (2.3–3.7)	0.5 (0.2–0.8)	1.9 (1.3–2.5)	1.3 (0.9–1.7)	–^††^
Florida	14.4 (13.5–15.3)	13.2 (12.3–14.1)	12.1 (11.3–13.0)	2.1 (1.7–2.5)	0.2 (0.1–0.3)	0.4 (0.2–0.5)	1.8 (1.5–2.2)	0.8 (0.6–1.0)
Georgia	16.8 (15.5–18.2)	14.7 (13.4–15.9)	13.4 (12.2–14.7)	2.1 (1.5–2.6)	–^††^	0.5 (0.3–0.8)	2.5 (1.9–3.0)	1.9 (1.4–2.4)
Hawaii	13.9 (12.2–15.6)	11.7 (10.1–13.2)	10.5 (9.0–12.0)	1.6 (1.0–2.1)	–^††^	–^††^	2.8 (1.9–3.6)	0.9 (0.5–1.4)
Idaho	17.4 (15.7–19.0)	14.7 (13.2–16.2)	13.3 (11.8–14.8)	2.1 (1.5–2.8)	–^††^	–^††^	3.9 (3.0–4.7)	2.2 (1.6–2.9)
Illinois	16.3 (15.1–17.4)	14.8 (13.7–15.8)	12.8 (11.8–13.8)	2.5 (2.0–3.1)	0.3 (0.1–0.5)	0.6 (0.3–0.8)	2.0 (1.6–2.4)	1.0 (0.7–1.3)
Indiana	22.5 (20.8–24.3)	20.3 (18.6–22.0)	18.9 (17.3–20.5)	3.1 (2.3–3.9)	–^††^	–^††^	3.1 (2.4–3.9)	2.2 (1.5–2.8)
Iowa	20.8 (18.9–22.6)	17.4 (15.7–19.1)	15.6 (14.0–17.2)	2.6 (1.8–3.3)	–^††^	–^††^	3.1 (2.4–3.9)	3.7 (2.8–4.6)
Kansas	22.1 (20.3–23.9)	19.5 (17.8–21.2)	17.6 (16.0–19.3)	3.0 (2.3–3.8)	0.5 (0.2–0.7)	0.9 (0.5–1.4)	3.5 (2.7–4.3)	2.9 (2.2–3.6)
Kentucky	26.2 (24.3–28.1)	22.4 (20.6–24.2)	21.1 (19.3–22.8)	2.5 (1.7–3.2)	–^††^	–^††^	3.7 (2.8–4.5)	3.8 (2.9–4.7)
Louisiana	21.6 (20.0–23.3)	19.5 (17.9–21.1)	18.6 (17.1–20.2)	2.1 (1.6–2.7)	–^††^	–^††^	2.5 (1.8–3.1)	2.4 (1.8–3.0)
Maine	18.6 (16.8–20.3)	17.8 (16.1–19.6)	16.1 (14.4–17.8)	2.6 (1.9–3.3)	0.8 (0.4–1.2)	–^††^	1.8 (1.2–2.5)	–^††^
Maryland	13.7 (12.3–15.2)	12.5 (11.1–13.9)	10.1 (8.8–11.4)	2.2 (1.6–2.8)	–^††^	–^††^	2.2 (1.5–3.0)	–^††^
Massachusetts	13.3 (11.9–14.7)	12.5 (11.1–13.9)	11.2 (10.0–12.5)	1.8 (1.3–2.4)	–^††^	–^††^	1.6 (1.1–2.1)	–^††^
Michigan	19.9 (18.5–21.3)	18.3 (17.0–19.7)	16.3 (15.0–17.6)	2.5 (1.9–3.1)	0.5 (0.3–0.7)	1.0 (0.5–1.4)	2.9 (2.3–3.4)	1.6 (1.1–2.1)
Minnesota	19.1 (17.6–20.7)	16.4 (14.9–17.8)	14.3 (12.9–15.7)	2.9 (2.2–3.6)	0.5 (0.2–0.8)	0.7 (0.3–1.1)	2.6 (1.9–3.2)	2.4 (1.8–3.0)
Mississippi	23.0 (21.3–24.7)	19.7 (18.1–21.3)	18.5 (17.0–20.1)	2.5 (1.9–3.1)	–^††^	–^††^	2.0 (1.5–2.6)	3.6 (2.8–4.5)
Missouri	20.7 (19.0–22.5)	18.0 (16.3–19.6)	16.9 (15.3–18.5)	2.0 (1.4–2.7)	–^††^	–^††^	3.1 (2.4–3.9)	2.0 (1.4–2.7)
Montana	21.8 (20.0–23.6)	18.5 (16.8–20.2)	16.3 (14.7–17.9)	2.8 (2.0–3.6)	0.9 (0.4–1.4)	–^††^	1.9 (1.3–2.5)	3.8 (3.0–4.6)
Nebraska	19.8 (18.0–21.6)	17.0 (15.3–18.7)	15.3 (13.7–17.0)	2.2 (1.5–2.8)	–^††^	–^††^	3.2 (2.3–4.0)	2.5 (1.8–3.2)
Nevada	16.6 (14.9–18.3)	15.6 (13.9–17.2)	14.1 (12.6–15.7)	1.4 (0.9–1.9)	–^††^	1.4 (0.7–2.1)	2.5 (1.9–3.2)	0.6 (0.2–0.9)
New Hampshire	17.3 (15.7–18.9)	15.9 (14.4–17.5)	14.1 (12.6–15.5)	2.4 (1.7–3.1)	–^††^	–^††^	2.2 (1.5–2.8)	1.0 (0.6–1.5)
New Jersey	12.2 (10.9–13.5)	11.9 (10.6–13.2)	10.1 (8.9–11.3)	2.3 (1.7–2.9)	–^††^	–^††^	1.5 (1.0–2.0)	–^††^
New Mexico	17.1 (15.5–18.8)	15.2 (13.6–16.8)	13.7 (12.2–15.2)	1.9 (1.2–2.5)	–^††^	–^††^	2.5 (1.8–3.1)	1.4 (0.9–1.9)
New York	14.5 (13.5–15.5)	13.8 (12.9–14.8)	12.2 (11.3–13.1)	2.2 (1.8–2.7)	0.3 (0.2–0.5)	0.6 (0.3–0.8)	1.6 (1.2–1.9)	0.6 (0.3–0.8)
North Carolina	20.4 (18.9–21.9)	17.7 (16.3–19.1)	16.0 (14.7–17.4)	2.8 (2.0–3.5)	–^††^	0.7 (0.3–1.0)	2.8 (2.2–3.4)	2.2 (1.6–2.8)
North Dakota	22.6 (20.7–24.5)	19.0 (17.2–20.7)	17.7 (16.0–19.4)	2.1 (1.5–2.7)	–^††^	0.9 (0.4–1.4)	2.2 (1.5–3.0)	4.9 (3.9–6.0)
Ohio	23.8 (22.5–25.2)	20.8 (19.6–22.1)	19.0 (17.8–20.2)	2.6 (2.1–3.2)	0.3 (0.1–0.5)	0.6 (0.3–0.9)	3.2 (2.6–3.8)	2.8 (2.2–3.3)
Oklahoma	23.8 (22.0–25.7)	19.7 (17.9–21.4)	18.5 (16.8–20.2)	2.5 (1.8–3.2)	–^††^	–^††^	3.6 (2.8–4.3)	4.3 (3.4–5.2)
Oregon	17.3 (15.7–19.0)	15.5 (13.9–17.1)	13.9 (12.4–15.4)	2.9 (2.1–3.7)	1.0 (0.6–1.5)	–^††^	3.6 (2.7–4.4)	2.1 (1.4–2.8)
Pennsylvania	20.5 (19.2–21.8)	18.1 (16.9–19.3)	15.8 (14.7–17.0)	3.2 (2.6–3.8)	0.4 (0.2–0.6)	–^††^	2.8 (2.2–3.4)	2.6 (2.1–3.1)
Rhode Island	15.5 (13.6–17.3)	14.3 (12.5–16.1)	11.6 (10.0–13.1)	2.6 (1.8–3.4)	–^††^	–^††^	2.5 (1.7–3.3)	–^††^
South Carolina	20.7 (19.0–22.4)	19.1 (17.5–20.7)	17.7 (16.1–19.2)	2.5 (1.8–3.2)	0.5 (0.2–0.7)	–^††^	2.8 (2.1–3.4)	1.2 (0.8–1.6)
South Dakota	23.0 (21.0–25.1)	19.5 (17.6–21.4)	18.8 (16.9–20.7)	2.3 (1.6–3.1)	–^††^	1.1 (0.6–1.7)	2.0 (1.4–2.7)	4.0 (2.9–5.0)
Tennessee	22.7 (21.1–24.4)	19.7 (18.1–21.3)	18.2 (16.7–19.7)	2.2 (1.6–2.8)	–^††^	–^††^	3.2 (2.5–3.9)	2.8 (2.1–3.5)
Texas	17.0 (16.2–17.9)	15.0 (14.1–15.8)	13.5 (12.7–14.3)	2.1 (1.7–2.4)	0.2 (0.1–0.3)	0.6 (0.3–0.8)	2.4 (2.1–2.8)	1.9 (1.6–2.2)
Utah	10.9 (9.5–12.4)	8.9 (7.6–10.2)	8.0 (6.8–9.2)	1.0 (0.5–1.5)	–^††^	0.9 (0.4–1.3)	3.1 (2.2–3.9)	1.3 (0.7–1.8)
Vermont	18.2 (16.5–19.9)	16.5 (14.9–18.1)	14.8 (13.3–16.3)	1.8 (1.2–2.4)	0.4 (0.2–0.7)	–^††^	1.8 (1.1–2.5)	1.8 (1.2–2.4)
Virginia	17.1 (15.7–18.5)	15.6 (14.2–16.9)	13.2 (12.0–14.5)	2.4 (1.8–3.0)	–^††^	1.1 (0.7–1.6)	2.3 (1.7–2.8)	1.4 (1.0–1.9)
Washington	16.8 (15.3–18.2)	14.8 (13.4–16.1)	12.8 (11.5–14.1)	2.7 (2.0–3.3)	0.8 (0.5–1.2)	0.8 (0.4–1.2)	2.5 (1.9–3.1)	2.2 (1.6–2.7)
West Virginia	26.9 (25.0–28.8)	23.1 (21.2–24.9)	21.7 (19.9–23.5)	1.9 (1.3–2.5)	–^††^	–^††^	3.8 (2.9–4.8)	4.8 (3.9–5.7)
Wisconsin	19.1 (17.5–20.7)	16.8 (15.3–18.3)	15.3 (13.9–16.7)	2.4 (1.7–3.0)	–^††^	–^††^	2.1 (1.5–2.6)	2.2 (1.5–2.9)
Wyoming	27.7 (25.6–29.7)	22.2 (20.3–24.1)	20.2 (18.4–22.0)	2.9 (2.1–3.8)	–^††^	–^††^	4.4 (3.5–5.2)	6.4 (5.2–7.6)

**Abbreviation**: CI = confidence interval.

* n = 163,920. Data were weighted to adjust for nonresponse and to yield representative estimates at the state level.

^†^ Persons who reported ever use of at least one of the six tobacco products assessed (cigarettes, cigars, regular pipe, water pipe, e-cigarette, and smokeless tobacco), and reported using the respective product “every day” or “some days” at the time of the survey.

^§^ Persons who reported having used cigarettes, cigars, regular pipe, or water pipe at least once during their lifetime and used “every day” or “some days” at the time of the survey.

^¶^ Persons who reported having smoked ≥100 cigarettes during their lifetime and smoked “every day” or “some days” at the time of survey.

** Persons who reported having used the respective product at least once during their lifetime and used “every day” or “some days” at the time of the survey. Cigars include regular cigars, cigarillos or little filtered cigars. Smokeless tobacco includes moist snuff, dip, spit, chew tobacco, snus, or dissolvable tobacco.

^††^ Estimates not presented because of relative standard error (RSE) ≥30%.

Cigarettes were the most common currently used tobacco product in all states and DC. The second most common currently used product in 23 states was e-cigarettes (range = 1.8% in Vermont to 3.9% in Idaho), cigars in 18 states and DC (1.7% in California to 3.5% in Alaska), and smokeless tobacco in nine states (3.6% in Mississippi to 6.4% in Wyoming).

Among persons reporting current use of any tobacco product, the proportion reporting concurrent use of two or more tobacco products ranged from 11.5% (Delaware) to 27.0% (Oregon). The proportion of current cigarette smokers reporting concurrent use of a noncigarette tobacco product ranged from 11.5% (Delaware) to 32.3% (Oregon) (Figure).

**FIGURE:**
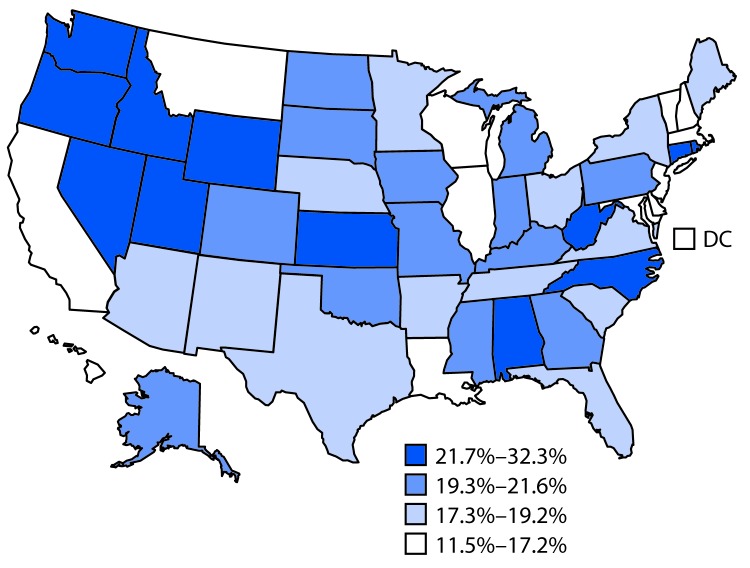
Proportion of current cigarette smokers* who reported concurrent use of noncigarette product^† ^— Tobacco Use Supplement to the Current Population Survey, United States, 2014–2015 Abbreviation: DC = District of Columbia. * Current cigarette smokers were persons who reported having smoked ≥100 cigarettes during their lifetime and smoked “every day” or “some days” at the time of survey (n = 23,232). Data were weighted to adjust for nonresponse and to yield representative estimates at the state level. The proportion of current cigarette smokers that reported concurrent use of a noncigarette tobacco product ranged from 11.5% in Delaware to 32.3% in Oregon. ^†^ Noncigarette tobacco products were five tobacco product types assessed in Tobacco Use Supplement to the Current Population Survey (TUS-CPS): cigars (regular cigars, cigarillos, or little filtered cigars), regular pipes, water pipes, electronic cigarettes, and smokeless tobacco products (moist snuff, dip, spit, chew tobacco, snus, and dissolvable tobacco).

## Discussion

Ever-use of any tobacco product by adults aged ≥18 years ranged from 27.0% (Utah) to 55.4% (Wyoming), and current use ranged from 10.2% (California) to 27.7% (Wyoming); nine of the 10 states with the highest prevalence of current use of any tobacco product were in the Midwest or South, and seven of the 10 states with the lowest prevalence were in the Northeast or West. Apart from regional and demographic characteristics, the differences across states in tobacco use might, in part, reflect differences in tobacco control and prevention interventions. For example, eight of the 10 states with the lowest prevalence of current use of any tobacco product have implemented policies that prohibit smoking in all indoor areas of workplaces, bars, and restaurants. In contrast, seven of the 10 states with the highest prevalence have no such comprehensive smoke-free laws.^††^ Continued implementation of proven population-based interventions, including increasing tobacco product prices, implementing and enforcing comprehensive smoke-free laws, warning about the dangers of tobacco use, and increasing barrier-free access to cessation services, can help reduce tobacco use (*1*,*4*).

Cigarettes were the most commonly used tobacco product, and nearly one in five current cigarette smokers concurrently used another form of tobacco. Among ever-users of each of the six tobacco products assessed, the proportion of current users was highest for cigarettes, followed by e-cigarettes. Given that most tobacco initiation occurs in adolescence and young adulthood (*6*), and product trial is a critical step in initiating and maintaining tobacco use (*7*), intensified efforts to prevent experimentation could reduce the likelihood of a lifetime of tobacco addiction. In light of the ever-changing tobacco control landscape, it is important to expand surveillance, policy, and programs to cover the range of tobacco products being marketed and used among youth and adults (*4*). For example, eight U.S. states and DC have expanded their comprehensive smoke-free laws to include e-cigarettes (*8*), and California and several U.S. cities have enacted policies prohibiting smokeless tobacco use in public sport arenas, which include 14 of 30 major league baseball stadiums.^§§^

The findings in this report are subject to at least three limitations. First, tobacco use was self-reported and might be underreported. Second, small sample sizes for some tobacco product types within certain states resulted in imprecise estimates that could not be presented. Finally, “ever-use” thresholds were characterized as ≥100 cigarettes versus ≥1 lifetime use for all other products; thus potentially underestimating both ever and current cigarette smoking.

Adoption of evidence-based measures across all states could help decrease tobacco use (*3**,**4*). Furthermore, continued tobacco surveillance at the national and state levels can help guide public health programs and policy (*4*,*8*).

**Summary**

**What is already known about this topic?**

Tobacco use is the leading cause of preventable morbidity and mortality in the United States. Despite recent declines in cigarette smoking prevalence, the tobacco product landscape has shifted to include emerging tobacco products, such as electronic cigarettes and water pipes.

**What is added by this report?**

Analysis of data from the 2014–2015 Tobacco Use Supplement to the Current Population Survey found that the prevalence of ever-use of any tobacco product ranged from 27.0% (Utah) to 55.4% (Wyoming). Current (every day or some days) use of any tobacco product ranged from 10.2% (California) to 27.7% (Wyoming). Cigarettes were the most common currently used tobacco product. Among current cigarette smokers, the proportion who currently used ≥1 other tobacco products ranged from 11.5% (Delaware) to 32.3% (Oregon). Eight of the 10 states with the lowest prevalence of current use of any tobacco product have implemented policies that prohibit smoking in all indoor areas of workplaces, bars, and restaurants; seven of the 10 states with the highest prevalence have no such comprehensive smoke-free laws.

**What are the implications for public health practice?**

Differences in tobacco product use across states underscore the importance of implementing comprehensive tobacco control and prevention interventions to reduce tobacco use and tobacco-related disparities, including comprehensive smoke-free policies, tobacco product price increases, anti-tobacco mass media campaigns, and barrier-free access to clinical smoking cessation resources.

## Footnotes

* Emerging tobacco products are noncigarette tobacco products that have gained increasing popularity and use within the U.S. market over the past decade.

† Tobacco Use Supplement to the Current Population Survey (TUS-CPS) is conducted in person or by proxy. The 2014–2015 survey was co-sponsored by the National Cancer Institute and the Food and Drug Administration Center for Tobacco Products and was administered as part of the U.S. Census Bureau’s Current Population Survey of the civilian, noninstitutionalized U.S. population. For the 2014–2015 iteration of the survey, 163,920 responses were self-reported (54,125 from July 2014, 56,652 from January 2015, and 53,143 from May 2015), and 67,476 were proxy-reported. Sensitivity analyses showed systematic differences between self- and proxy-responses in relation to key demographic characteristics, especially for younger adults. To reduce likelihood of misclassification bias, proxy responses were excluded from these analyses. The TUS-CPS self-response weights derived by the Census Bureau count proxy respondents as “nonrespondents” when an analysis is conducted only with self-respondents. https://cancercontrol.cancer.gov/brp/tcrb/tus-cps/.

§ The following smokeless tobacco products were combined together and analyzed as a class of products: moist snuff, dip, spit, chew tobacco, snus, or dissolvable tobacco. Use of smokeless tobacco product was assessed with separate questions: “Have you ever used any of the following even one time? Smokeless tobacco, such as moist snuff, dip, spit, chew tobacco or snus,” “Have you ever used dissolvable tobacco even one time?” and whether the respondent used the respective product “every day” or “some days” at the time of survey. Participants who had at least one missing response to these questions were excluded from the analysis (1.4% [2,356] of respondents for ever-use; 1.4% [2,373] of respondents for current use).

¶ Participants who had at least one missing response to any of the assessed tobacco product type questions were excluded from the analysis (1.7% [2,734] of respondents for ever-use; 1.8% [2,875] of respondents for current use).

** Participants who had at least one missing response to any of the combustible tobacco product type questions were excluded from the analysis (1.5% [2,489] of respondents for ever-use; 1.6% [2,645] of respondents for current use).

††
https://www.cdc.gov/mmwr/volumes/65/wr/mm6524a4.htm .

§§
http://tobaccofreebaseball.org/news-coverage/ .
